# Epidemic characteristics and effectiveness of vaccine intervention on rotavirus infection: a real-world observational study in Zhejiang Province, China

**DOI:** 10.3389/fpubh.2025.1596899

**Published:** 2025-05-09

**Authors:** Ziping Miao, Yuxia Du, Anqi Dai, Mengya Yang, Can Chen, Rui Yan, Jian Gao, Yijuan Chen, Kexin Cao, Daixi Jiang, Xiaobao Zhang, Xiaoyue Wu, Mengsha Chen, Yue You, Wenkai Zhou, Dingmo Chen, Jiaxing Qi, Shiyong Zhao, Xianyao Lin, Shigui Yang, RIDPHE Group, Shigui Yang, Xudong Zhou, Peige Song, Ning Zhang, Hao Lei, Junfang Xu, Jianbing Wang

**Affiliations:** ^1^Department of Communicable Diseases Control and Prevention, Zhejiang Provincial Center for Disease Control and Prevention, Hangzhou, China; ^2^Department of Epidemiology and Biostatistics, School of Public Health, Department of Emergency Medicine, Second Affiliated Hospital, The Key Laboratory of Intelligent Preventive Medicine of Zhejiang Province, Zhejiang University School of Medicine, Hangzhou, China; ^3^Department of the Institution for Drug Clinical Trials, Quzhou People's Hospital, Quzhou, Zhejiang, China; ^4^Department of Immunization Program, Zhejiang Provincial Center for Disease Control and Prevention, Hangzhou, China; ^5^Department of Microbiology, Zhejiang Provincial Center for Disease Control and Prevention, Hangzhou, China; ^6^Department of Infectious Diseases, Hangzhou Children’s Hospital, Hangzhou, China

**Keywords:** rotavirus infection, incidence, epidemiological trends, rotavirus vaccines, vaccine effectiveness

## Abstract

**Background:**

Rotavirus infection, the most common cause of infant infectious diarrhoea and related deaths worldwide, has imposed a high disease burden in China, especially in Zhejiang Province. This study described the overall epidemiological characteristics and trends of reported rotavirus infections in Zhejiang Province from 2005 to 2022 and evaluated the effectiveness of rotavirus vaccines on the incidence of rotavirus infection.

**Materials and methods:**

Data on reported cases of rotavirus infection from 2005 to 2022 were extracted from the China Disease Prevention and Control Information System. Information on rotavirus vaccination was obtained from the Zhejiang Provincial Viral Diarrhoea Surveillance Site in 2022. Join-point regression, spatial and temporal aggregation analysis, and an age-period-cohort model were used to explore the epidemiological trends of rotavirus infection. Interrupted time series analysis and an overdispersed Poisson model were used to quantify the effectiveness of rotavirus vaccines.

**Results:**

The average age-standardized reporting incidence rate (ASRIR) of rotavirus infection in Zhejiang Province was 38.58/100,000, particularly in children aged 0–2 years, who had the highest average annual incidence of 951.63/100,000. The annual ASRIR of all ages showed a significant upward trend before 2017 (average percentage change [APC] = 21.64%) and then decreased significantly (APC = −23.02%). However, in children aged 6–19 years, the annual incidence presented a sustained and significant upward trend over time. The rotavirus infection peak showed a seasonal drift in Zhejiang Province, shifting from November before 2014 to January after 2014. Spatiotemporal aggregation revealed two clusters. The spatio-temporal scanning found two spatio-temporal aggregation areas, the first level spatio-temporal aggregation area was distributed in Hangzhou, Jiaxing and Huzhou, and the second level spatio-temporal aggregation area was Lishui. The age-period-cohort model indicated that the risk of rotavirus infection was primarily concentrated in children aged 0–4 years. The vaccine effectiveness (VE) of rotavirus vaccines was 71.62% (95% confidence interval [CI]: 45.21–86.05%) in children aged 2–59 months, in which the VE of the human-bovine reassortant pentavalent vaccine (RV5) was 91.31% (95% CI: 74.39–97.97%). Since the implementation of RV5 vaccination in September 2018, the number of cases of rotavirus infection per month has decreased by 3,061 (65.27%) in Zhejiang Province.

**Conclusion:**

The disease burden of rotavirus infection in Zhejiang Province was high, especially in children. Rotavirus vaccination have significantly reduced the incidence rate of rotavirus infection. Therefore, the prevention of infectious diarrhoea should be further strengthened, especially through increased coverage with the rotavirus vaccine.

## Introduction

Studies based on the Global Burden of Disease Study 2016 indicated that diarrhoea was the eighth leading cause of death for all ages and the fifth leading cause of death for children aged 0–4 years, placing a considerable burden on public health (1, 2). Moreover, rotavirus infection has consistently been the leading cause of diarrhoea-related deaths in all age groups, accounting for 25.50% of deaths in 1990 and 19.45% in 2019 (3). Rotavirus infection in children under 5 years old has caused 124 million clinical visits, 9 million hospitalizations, and 1.3 million deaths worldwide each year (4).

As rotavirus is the primary pathogen involved in infectious diarrhoea, rotavirus infections and related deaths have occurred predominantly in developing countries, particularly in Africa, Oceania, and South Asia (3). In 2013, India, Nigeria, Pakistan, and Congo accounted for 49% of the estimated global rotavirus-related deaths (5). China is also among the countries with a high disease burden of diarrhoea (6) and has the highest annual social cost of treating rotavirus infections ($365 million) among Asian countries (7). In a survey of 32 provincial-level administrative regions in China, Zhejiang Province ranked second in terms of the number of rotavirus infections (133,000 cases) among children under 5 years old from 1994 to 2013 (8), indicating the need for further attention to rotavirus infection in Zhejiang Province. In order to reduce the burden of rotavirus infection, various preventive measures like rotavirus vaccine, probiotics, improvements in levels of hygiene and the quality of food and water were adopted (9–12). Among them, rotavirus vaccine is considered to be the most effective method to prevent rotavirus infection, because a single infection can enhance immunity and reduce an individual’s chance of future infection (8). As early as 2009, the World Health Organization (WHO) Strategic Advisory Group of Experts recommended promoting rotavirus vaccines in all regions worldwide, especially in developing countries and countries with high rotavirus-related mortality rates (13). There are currently two main types of rotavirus vaccines available in China. One is the monovalent rotavirus vaccine (MRV), available in China since 2000, and the other is the human-bovine reassortant pentavalent vaccine (RV5), available in China since September 2018. The effectiveness (VE) of the monovalent rotavirus vaccine is controversial (14–16), but the VE of the RV5 vaccine against moderate or severe gastroenteritis caused by rotavirus infection is consistently high (17). However, rotavirus vaccines have not yet been included in China’s national immunization plan; thus, vaccination coverage remains low.

Given the limited understanding of the long-term epidemiological patterns of rotavirus infection in Zhejiang Province, this study aimed to characterise the temporal trends, seasonal variations and age-specific burden of rotavirus infections from 2005 to 2022. In addition, we assessed the real-world effectiveness of the rotavirus vaccine, using routine surveillance data from hospitalised children aged 2–59 months. These findings are expected to provide epidemiological evidence to inform targeted strategies for rotavirus control and vaccine implementation in Zhejiang Province and similar settings.

## Materials and methods

This study obtained data from the China Disease Prevention and Control Information System, the Zhejiang Provincial Bureau of Statistics, the National Statistical Yearbook, and the Viral Diarrhoea Surveillance Site in Zhejiang Province. Detailed information can be found in Supplementary material 1.

### Test negative case–control study

Study population: Data on diarrhoea cases were collected from 4 monitoring locations (Bin, Jiang District, Gongshu District, Huzhou City, and Lishui City) in Zhejiang Province. Detailed information can be found in Supplementary material 1.

The inclusion and exclusion criteria were as follows: (1) children hospitalised for diarrhoea or diarrhoea symptoms during hospitalisation who met the clinical criteria for diarrhoea and stayed in the hospital for ≥ 24 h; (2) children with sufficient fresh stool samples for RV testing; (3) children aged 2–59 months who were eligible to receive a rotavirus vaccine; (4) children with a detailed rotavirus vaccination record; and (5) children who received rotavirus vaccination ≥14 days before the onset of diarrhoea symptoms.

Research method: A negative case–control study design was used to evaluate the effectiveness of rotavirus vaccines in preventing rotavirus-related diarrhoea in infants and young children by comparing the Odds Ratio (OR) values of rotavirus vaccination before diarrhoea onset between the case group (rotavirus-positive diarrhoea patients) and the control group (rotavirus-negative diarrhoea patients). We further performed sensitivity analyses adjusting for age, sex, and region.

### Statistical analysis

#### Epidemiological trend analysis

A joinpoint regression model was used to examine the reported incidence trends of rotavirus infection by age groups (0–2 years, 3–5 years, 6–19 years, 20–59 years old, and ≥ 60 years) and sex from 2005 to 2022. The annual percentage changes (APCs) and their 95% confidence intervals (CIs) were calculated for each trend segment (18). A Z test was used to assess whether the APCs were significant (*p* < 0.05). The analysis was conducted via Joinpoint (version 4.9.0.0) (19). Detailed information can be found in Supplementary material 2.

To better display the seasonal changes of rotavirus infections, we divided the monthly reported rotavirus infection data in Zhejiang Province from 2005 to 2022 and loaded them into EPIPOI software. Quadratic polynomials were used to detrend the time series data, and the periodic annual function (PAF) was generated by summing the annual, semi-annual, and quarterly harmonics obtained from the fourier decomposition (20). We extracted the timing of the primary peak, representing the period during which the maximum intensity of the disease burden typically occurs (20). Further subgroup analysis was performed according to age groups (0–2 years, 3–5 years, 6–19 years, 20–59 years, and ≥ 60 years).

#### Age-period-cohort model analysis

The trend of disease incidence may be influenced by age effects, period effects, and birth cohort effects. Thus, the age-period-cohort model was used to separate these three influences. The intrinsic estimator (IE) (21) was used to avoid collinearity among age, period, and birth cohorts.

The calculation formula of the age-period-cohort model was as follows:


$$\ln\left(Y_{ij}\right)=\pi +\alpha_{i}+\beta_{j}+\delta_{k}$$

Where $Y_{ij}$ represents the reported incidence of rotavirus infection, π represents the intercept, $\alpha_{i}$ represents the age effect of the i age group, $\beta_{j}$ represents the period effect of the j period group, and $\delta_{k}$ represents the cohort effect of the k birth cohort group (22).

First, we divided the entire sample into 16 age groups based on 5-year intervals (from 0–4 to 75–79), 4 period groups (from 2005–2009 to 2020–2022; owing to the lack of data for 2023–2024, the last period represented the average incidence from 2020 to 2022), and 19 birth cohort groups (from 1926–1930 to 2016–2020). The rate ratio (RR) for each group in the age-period-cohort model results represented a multiple of the incidence risk for that group compared with the overall incidence risk for the entire group. The RR was calculated as follows: $RR=\exp^{\left(\mathrm{coef}\text{.}\right)}$.

Since young children are at high risk of rotavirus infection, we conducted an age-period-cohort model analysis on children aged 0–4 years. The children were divided into five age groups, each representing a 1-year age interval. Thus, there were 18 period groups based on 1-year intervals (from 2005 to 2022) and 22 birth cohort groups (from 2001 to 2022) (23). The age-period-cohort model analysis was conducted on the web version of the age-period-cohort model analysis tool,^1^ and longitudinal age effects revealed age-associated effects on rotavirus infection (24).

#### Spatial and temporal aggregation analysis

We used spatiotemporal scan statistics performed with SatScan (version 9.5) to explore the space–time clustering of reported rotavirus infections in Zhejiang Province (25). A dynamic space–time two-dimensional cylinder scanning window was constructed to scan each location (city) within the study area. The actual and theoretical incidence numbers inside and outside the scanning window were used to calculate the log-likelihood ratio (LLR) and RR. The cluster was classified according to the LLR value (26). The significance level was set at *p* < 0.05. Detailed information can be found in Supplementary material 3.

#### Interrupted time series analysis

An interrupted time series analysis (ITSA) model was constructed to analyse the changes in the reported annual rotavirus infection incidence in Zhejiang Province before (2005–2018) and after (2018–2022) the intervention with the RV5 (27, 28). Detailed information can be found in Supplementary material 4.

In addition, we estimated the expected monthly incidence of rotavirus infection in September 2018 without the RV5 intervention using an overdispersed Poisson model (29, 30). Detailed information can be found in Supplementary material 4.

The R (4.2.2) software was used to fit and analyse the above models.

#### Other statistical analyses

Excel 2016 was used to clean and organise reported cases of rotavirus infection, as well as case information and specimen testing data provided by sentinel hospitals. Spearman’s correlation was used to analyse the association of incidence rates among different age groups. Logistic regression analysis was used to obtain the OR value and 95% CI of rotavirus vaccination before the onset of diarrhoea between the case and control groups. The VE calculation formula was as follows: VE = (1-OR) × 100% (31). Sensitivity analysis was further performed on age, sex, and location, and the test level was *p* = 0.05.

## Results

### Epidemiological trends of rotavirus infection in Zhejiang Province from 2005 to 2022

**Figure 1:**
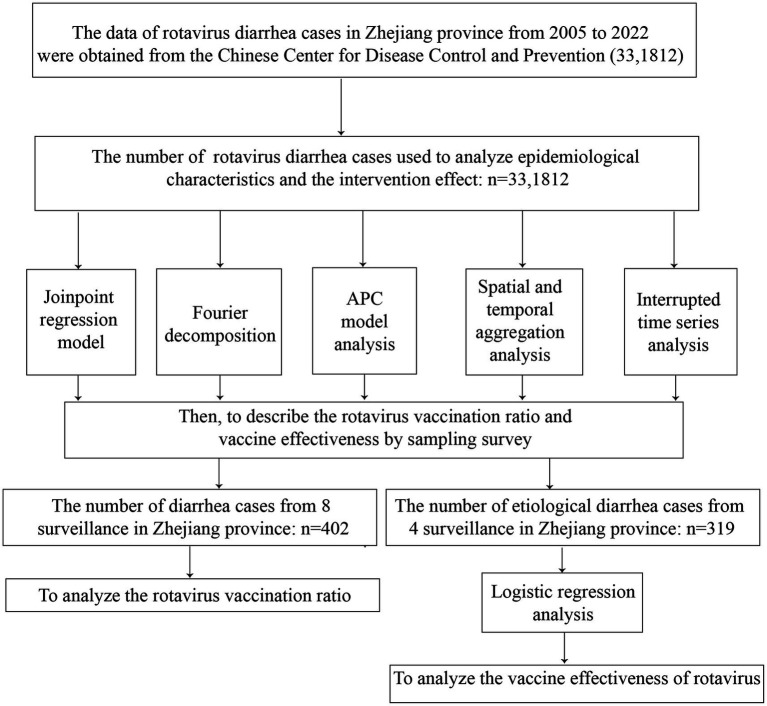
Flow chart.

**Figure 2:**
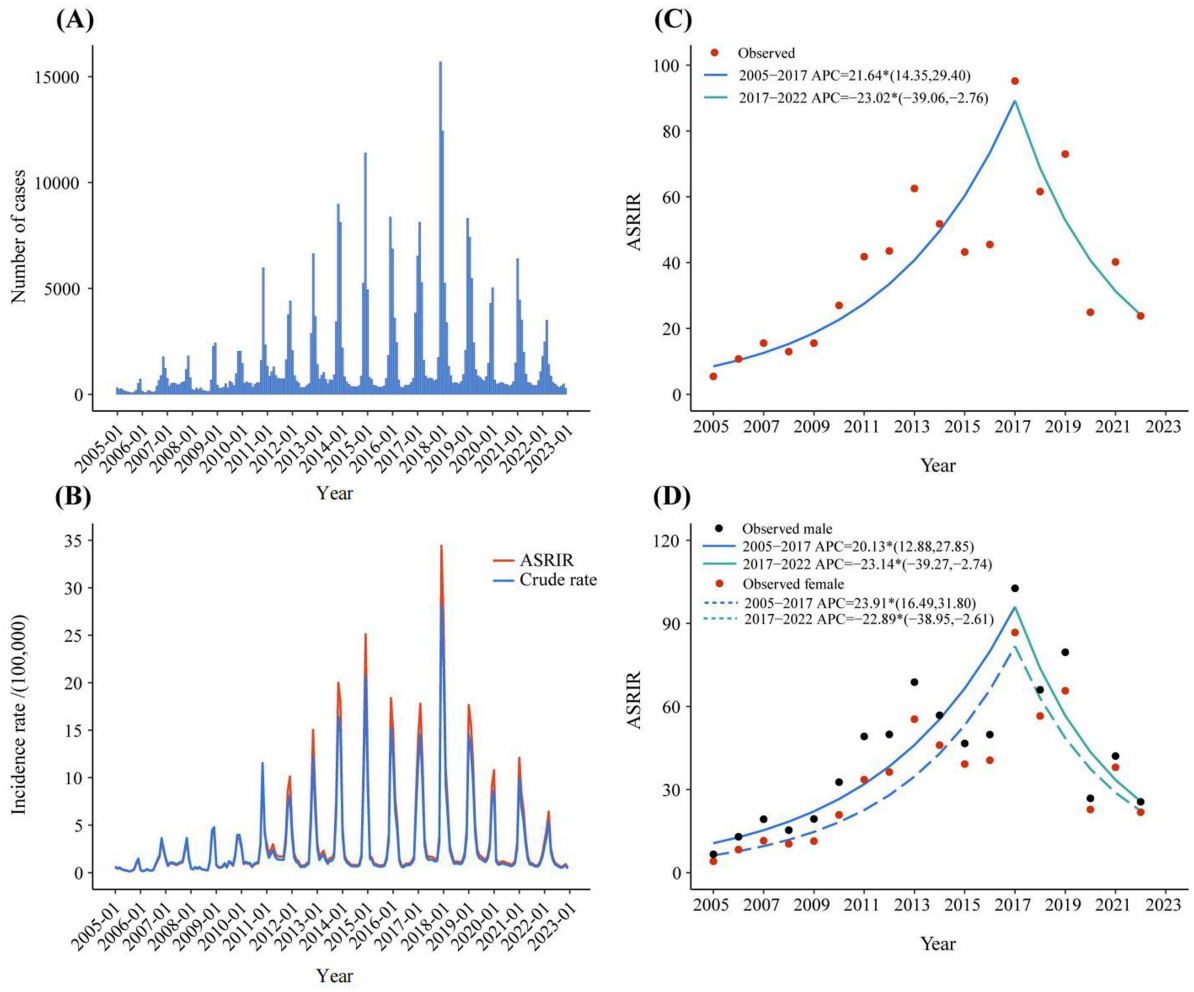
The reported incidence and trend of rotavirus infection in Zhejiang province from 2005 to 2022. **(A)** The monthly reported number of cases of rotavirus infection in 2005–2022. **(B)** ASRIR and unadjusted monthly reported incidence of rotavirus infection in 2005–2022. **(C)** The joinpoint analysis of rotavirus infection in 2005–2022. **(D)** The joinpoint analysis of rotavirus infection in different genders in 2005–2022. ASRIR: Age-standardized reporting incidence rate.

The flow chart was shown in Figure 1. A total of 33,1812 rotavirus infection cases were reported in Zhejiang Province from January 1, 2005, to December 31, 2022. The average annual age-standardized reporting incidence rate (ASRIR) was 38.58/100,000. The ASRIR of rotavirus infection was high from 2014 to 2019, especially in 2017 (95.18/100,000). The ASRIR of rotavirus infection demonstrated clear seasonality and periodicity (Figures 2A,B). The ASRIR of rotavirus infection presented a significant upward trend in Zhejiang Province before 2018 (APC = 21.64%, *p* < 0.05), followed by a significant downward trend after 2018 (APC = −23.02%, *p* < 0.05) and the male and female trends were the same (Figures 2C,D).

**Figure 3:**
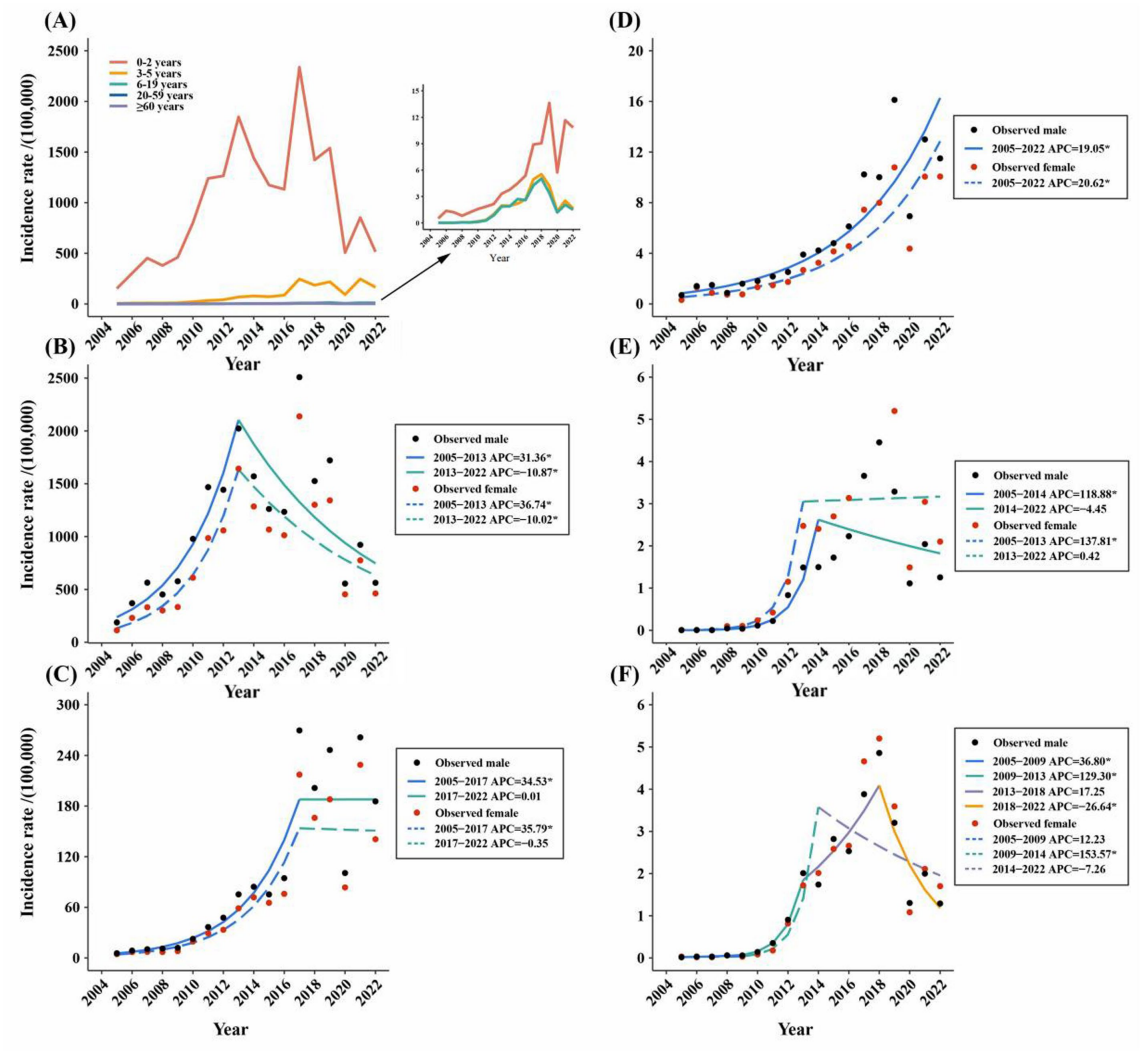
**(A)** Incidence rate of reported rotavirus infection in different age groups in Zhejiang province from 2005 to 2022. **(B–F)** The joinpoint analysis of reported rotavirus infection incidence in different age groups in Zhejiang province from 2005 to 2022: **(B)** 0–2 years old. **(C)** 3–5 years old. **(D)** 6–19 years old. **(E)** 20–59 years old. **(F)** ≥ 60 years old.

According to the age-stratified analysis, the highest average annual reported incidence was found in the 0-2-year age group, which was 951.63/100,000. The age group over 60 years had the lowest average annual incidence rate (1.76/100,000) (Figure 3A). The join-point regression results revealed that the reported incidence of both males and females in the age groups of 0–2 years, 3–5 years, 20–59 years, and over 60 years initially increased but then decreased or remained stable (Figures 3B,C,E,F). Notably, the reported incidence among males and females in the 6-19-year age group consistently and significantly increased from 1990 to 2019, with APCs of 19.05 and 20.62, respectively (Figure 3D). In addition, the reported incidence in the age groups of 20–59 years and over 60 years were higher in females than in males, whereas the reported incidence in males were all higher than those in females in the other age groups in 2022 (Figure 3). Finally, we analysed the correlation between the incidence rates of rotavirus infection in different age groups. We found a significant positive correlation between the incidence rates in each age group (Supplementary material 5).

**Figure 4:**
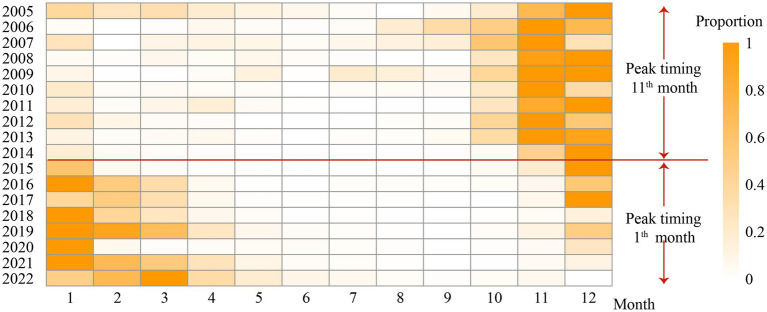
The heat-map of reported incidence of rotavirus infection in the whole population from January 2005 to December 2022. The color bar indicates the intensity of incidence, counts from high (orange) to low (white). Monthly incidence counts were standardized for each year and shown as the proportion of the maximum number of cases in a month for that period (hence, months with the maximum number of cases for a given season were assigned the value 1).

Figure 4 shows the seasonal peak shift in reported cases of rotavirus infection in Zhejiang Province. For all ages, the peak before 2014 occurred in November, whereas the peak after 2014 occurred in January. In the 0-2-year age group, the change in peak timing was mirrored across all ages. For the groups aged 3–5 years and 20 years and older, the peak timing before 2014 occurred in December, whereas the peak timing after 2014 occurred in January. In the group aged 6–19 years, the peak before 2014 occurred in December, whereas the peak after 2014 occurred in February (Supplementary material 6). The peak timing for all age groups was delayed by over a month.

**Figure 5:**
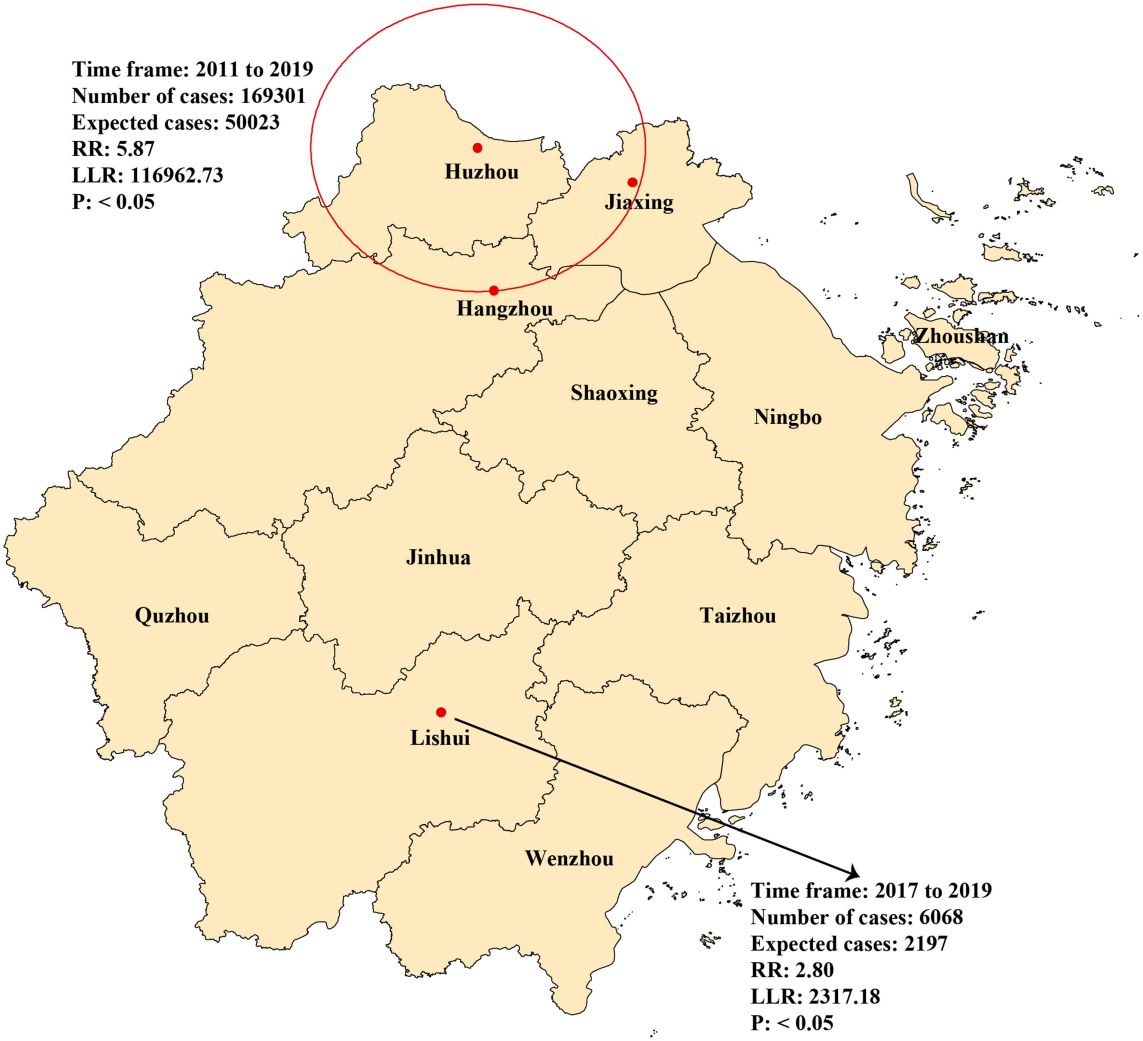
Spatial and temporal aggregation of rotavirus infection in Zhejiang province from 2005 to 2022.

The observed number of cases in these regions was 169,301, and the number of expected cases was 50,023 (RR = 5.87, LLR = 116,962.73, *p* < 0.05). The secondary spatiotemporal aggregation area was Lishui, with cases concentrated between 2017 and 2019. The actual number of cases reported in the region was 6,068, and the number of expected cases was 2,197 (RR = 2.80, LLR = 2,317.178, *p* < 0.05) (Figure 5).

**Figure 6:**
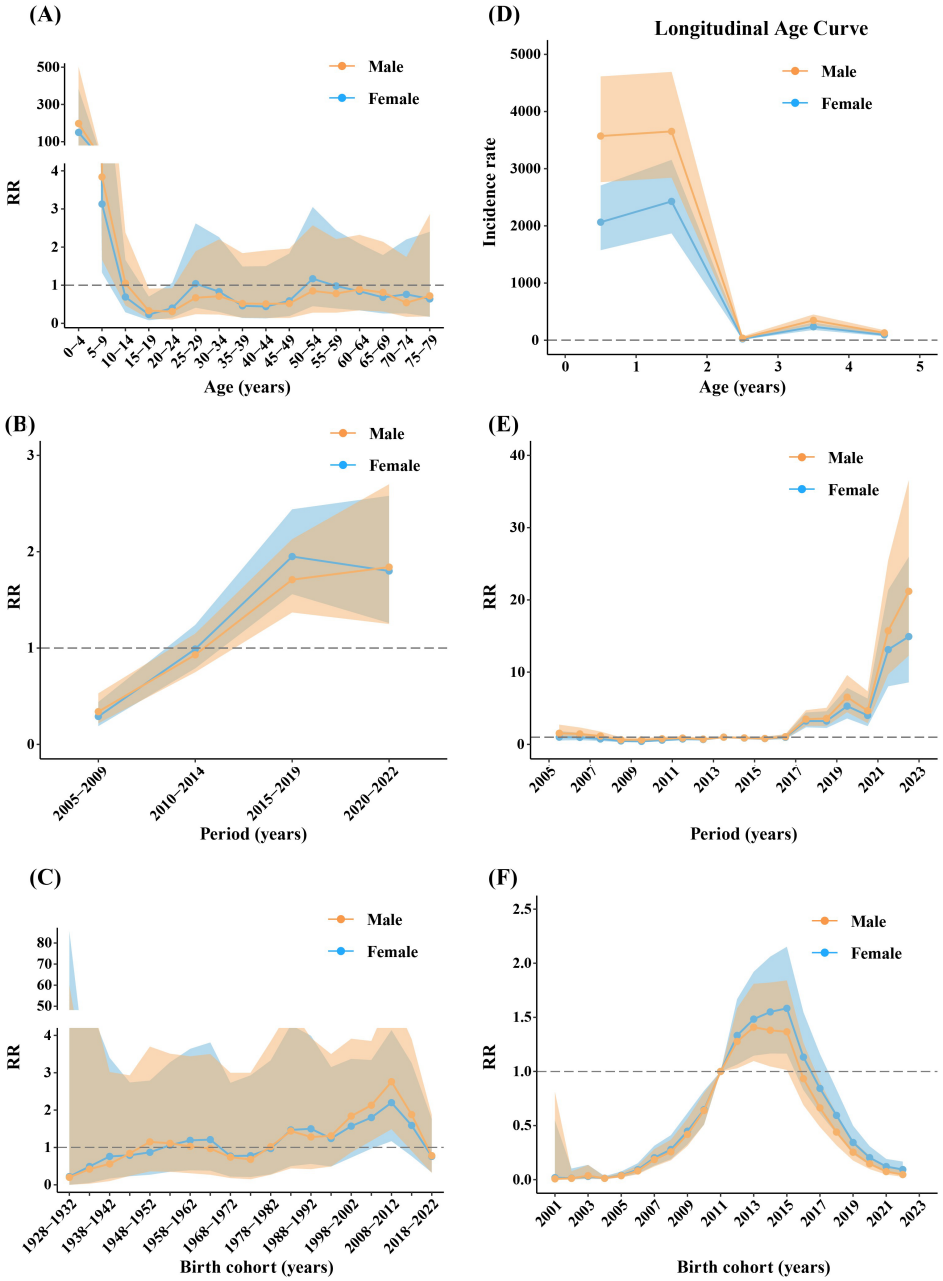
The age **(A,D)**, period, **(B,E)** and cohort **(C,F)** effects of different genders across all ages **(A–C)** and 0–4 year age group **(D–F)**. Shadows of different colors represent 95% confidence intervals for the corresponding gender.

The effects of age on the reported incidence of rotavirus infection in males and females were similar, generally decreasing with increasing age, although most RRs were not statistically significant. The group aged 0–4 years had the highest RR (male: 197.63, 95% CI: 77.94–501.12; female: 149.81, 95% CI: 58.80–381.72) (Figure 6A). The RRs of period effects in males and females were significantly less than 1 during 2005–2009, whereas those were significantly greater than 1 between 2015 and 2019 and 2020–2022 (Figure 6B). The RRs of birth cohort effects in males and females were mostly not statistically significant. Males and females born between 2008 and 2012 had the highest risk, with RR values of 2.76 (95% CI: 1.49–5.10) and 2.20 (95% CI: 1.17–4.14), respectively (Figure 6C).

The effects of age on the reported incidence of rotavirus infection in males and females in the 0–4 age group were similar, generally decreasing with age. The incidence risk in males was notably higher than that in females. The incidence rate was especially high in the 1-2-year-old group (male: 3652.42, 95% CI: 2842.60–4692.94; female: 2427.9922, 95% CI: 1869.16–3153.90) (Figure 6D). The RRs of period effects in males and females were very similar, remaining stable before 2016, showing an increasing trend starting in 2017, especially after 2021, and peaking in 2022 (male: 21.19, 95% CI: 12.29–36.54; female: 14.92, 95% CI: 8.58–25.94) (Figure 6E). The cohort effects in males and females were similar, with RRs increasing before 2015 and decreasing after 2015. The incidence risk was significantly greater in the 2012–2015 birth cohorts (Figure 6F).

### Sampling survey of rotavirus vaccination ratio and its intervention effect on the reported incidence of rotavirus infection

**Figure 7:**
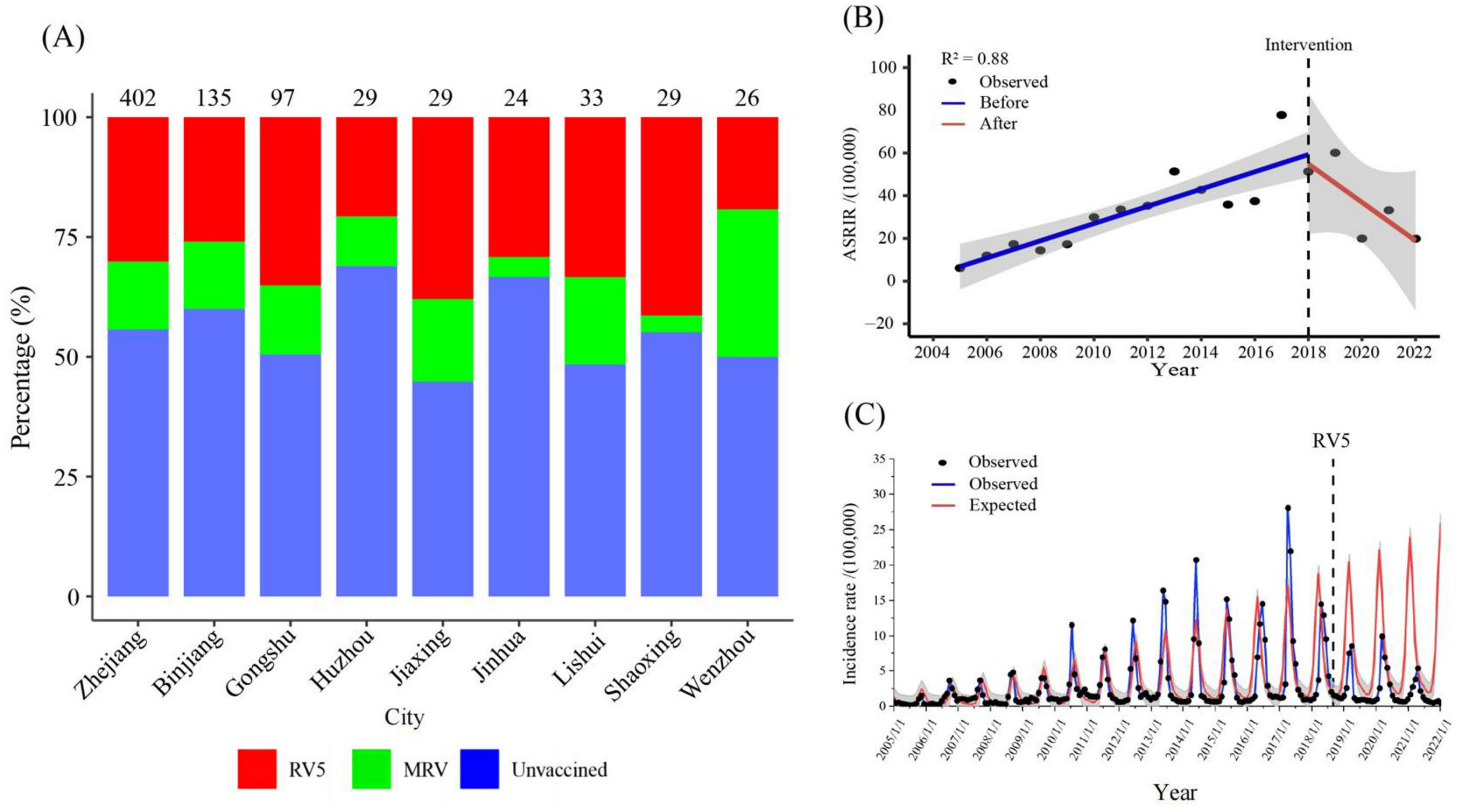
**(A)** Percentage of children aged 2–59 months hospitalized for diarrhoea surveyed for rotavirus vaccination at eight surveillance locations in 2022. **(B)** Interrupted time series analysis of reported incidence of rotavirus infection in Zhejiang province from 2005 to 2022. **(C)** The expected reported incidence of rotavirus infection in Zhejiang province without RV5 intervention and the observed reported incidence. RV5: Human-bovine reassortant pentavalent vaccine; ASRIR: Age-standardized reporting incidence rate.

In 2022, a survey was conducted at eight monitoring sites with children aged 2–59 months hospitalised due to infectious diarrhoea. The results revealed that the total rotavirus vaccination ratio of rotavirus vaccines was 44.28% in diarrhoea patients, for whom the vaccination ratio of RV5 was 30.10%, and the vaccination ratio of the monovalent rotavirus vaccine was 14.18%. The total rotavirus vaccination ratios in Binjiang Hangzhou, Gongshu Hangzhou, Huzhou, Jiaxing, Jinhua, Lishui Shaoxing, and Wenzhou were 40.00, 49.48, 31.03, 55.17, 33.33, 51.52, 44.83, and 50.00%, respectively (Figure 7A).

The ASRIR of rotavirus infection from 2005 to 2018 showed a significant increasing trend ($\beta_{1}$= 4.10, *p* < 0.05), and the reported incidence of rotavirus infection from 2018 to 2022 showed a decreasing trend ($\beta_{1+}\beta_{3}$= − 10.70) (Figure 7B). According to age subgroup analyses, the incidence rate in the 6-19-year-old group significantly increased from 2005 to 2018 ($\beta_{1}$= 0.51, *p* < 0.05). In contrast, after the implementation of rotavirus vaccine intervention, the slope change was not significant ($\beta_{3}$= − 0.67, *p* = 0.14) (Supplementary material 7). Moreover, it was predicted that without RV5 intervention, the expected average monthly number of cases of rotavirus infection after September 2018 would be 4,690, whereas the actual average monthly number would be 1,629. However, the reported average monthly number decreased by 3,061 cases, a decrease of 65.27% in reported rotavirus infections (Figure 7C).

In 2022, four surveillance locations were tested for rotavirus aetiology, and we further analysed the effectiveness of rotavirus vaccines. According to the inclusion and exclusion criteria, 282 diarrhoea patients were included (Supplementary material 8). Among the 282 children with diarrhoea, 63 (22.34%) were confirmed to be rotavirus-positive. There was no significant difference in age or sex between the two groups (*p* > 0.05). Fourteen children (22.22%) with rotavirus-related diarrhoea received rotavirus vaccines, of whom 11 received MRV, and 3 received RV5. A total of 107 control children (48.86%) were vaccinated with rotavirus vaccines, of whom 29 were vaccinated with MRV, and 78 were vaccinated with RV5 (Supplementary material 9). Table 1 indicates that the VE of rotavirus vaccines was 71.62% (95% CI: 45.21–86.05%) in children aged 2–59 months with diarrhoea, in which the VE of RV5 was 91.31% (95% CI: 74.39–97.97%).

**Table 1:** The positive detection of rotavirus infection among hospitalized children with diarrhea under the age of 5 and the vaccination status of different types of rotavirus vaccines in 2022.

Vaccination statuses	Rotavirus diarrhea (*n* = 63)	Other diarrhea (*n* = 219)	Crude VE (95% CI)	*p*-value	Adjusted^*^ VE (95% CI)	*p*-value
Rotavirus vaccines^†^						
Vaccinated	14	107	70.09 (44.02–84.88)	< 0.05	71.62 (45.21–86.05)	< 0.05
Unvaccinated	49	112	Ref		Ref	
Pentavalent vaccine						
Vaccinated	3	107	91.21 (74.92–97.91)	< 0.05	91.31 (74.39–97.97)	< 0.05
Unvaccinated	49	112	Ref		Ref	

† Include pentavalent vaccine and monovalent rotavirus vaccine.

* Adjusted for age, gender and area.

## Discussion

This study presented the epidemiological trends and distribution characteristics of 331,812 reported cases of rotavirus infection in Zhejiang Province during the 18-year period, investigated the rotavirus vaccination rate in children aged 2–59 months admitted to sentinel hospitals due to diarrhoea in 2022, and quantified the effectiveness of the RV5 in rotavirus prevention and control. The results revealed that the incidence rate of rotavirus infection in Zhejiang Province significantly increased before 2017. Although the incidence rate of rotavirus infection in young children decreased after 2013, they were still at high risk. Moreover, the peak month of rotavirus infection in Zhejiang Province shifted backward, moving from November in the pre-2014 period to January in the post-2014 period. Two spatiotemporal aggregation regions were discovered, and high-risk age, period, and birth cohorts of rotavirus infection were identified. In addition, the RV5 was confirmed to be an effective intervention. Our results provide a scientific basis for decision-makers to better prevent and control rotavirus infection in the future.

The significant decline in the ASRIR from 2017 to 2022 may be related to the implementation of RV5 in young children because rotavirus is transmitted through contact transmission (especially faecal-oral transmission) (32, 33) (Figure 2). Thus, RV5 could reduce the incidence of rotavirus infection in young children and decrease contact transmission in families. In terms of age groups, the incidence rate in the group aged 6–19 years increased from 2005 to 2022 (Figure 3), which may be attributed to the fact that adolescents aged 6–19 years are not the target population for the RV5 vaccine. Consequently, the introduction of the RV5 in China in 2018 had no impact on the incidence rate of rotavirus among adolescents. The decline in incidence rates of other age groups was primarily due to the decline in the incidence rate in young children. Because rotavirus is transmitted mainly via the faecal-oral route, with the possibility of contact transmission, young children are the primary contact sources for adults and the older adult (34). This also suggests that the continuing increase in incidence among adolescents aged 6–19 warrants further investigation, and an in-depth analysis of the causes is needed. In addition, the incidence rates were generally higher among women than men in 2022 in the 20–59 years and over 60 years groups (Figure 3), possibly because adult women have more frequent contact with infants and young children.

Diarrhoea usually exhibits specific spatiotemporal clusters influenced by sociodemographic variables, such as personal hygiene, the environment, and climate change (18, 35–37). The spatiotemporal aggregation of rotavirus infection in Zhejiang Province during the studied period could be divided into two stages. From 2011 to 2019, a first-level spatiotemporal aggregation area was discovered in Hangzhou, Huzhou, and Jiaxing (Figure 5). These areas are developed in Zhejiang Province, which has a higher population density and a large floating population, which may have poor living conditions and lower hygiene awareness. From 2017 to 2019, Lishui, the secondary spatiotemporal aggregation area (Figure 5), had a very low GDP in Zhejiang Province, indicating limited healthcare resources, living environments, and disease prevention and control measures. These findings highlight that rotavirus incidence trend may be affected by numerous factors other than vaccine. Lifestyle changes and immune status play significant roles in shaping these trends, and understanding them is crucial for effective disease prevention and control. Improved hygiene practices can have a notable impact on rotavirus infection rates. For instance, enhanced hand hygiene programs, especially including hand sanitizer, can reduce the spread of the virus in young children (38). Additionally, in the older adult, immune senescence occurs, which means the immune system becomes less efficient, which can affect the immune response to pathogen. This age-related decline in immune function can make them more susceptible to rotavirus infection (39). Consequently, optimal rotavirus control requires an integrated strategy that combines vaccination with targeted interventions such as hygiene education in urban centers and healthcare system strengthening in disadvantaged areas, providing a comprehensive approach for sustainable disease management in Zhejiang Province and similar developing regions.

Rotavirus infection in the province exhibited a seasonal peak drift phenomenon (Figure 4), a trend also observed in South China. Compared with 2007 to 2010, the peak incidence of RV infection in South China from 2013 to 2015 was delayed by 7 weeks (40). However, although China has a nationwide RV surveillance network, we currently only have the data on reported rotavirus infection cases in Zhejiang Province and do not have relevant data to assess changes in reported incidence, such as vaccination coverage. Therefore, it was impossible to determine whether the shift in seasonality was due to vaccination or other factors. The age-period-cohort model analysis revealed that for children aged 0–4 years, the incidence risk significantly increased after 2016, particularly after 2021, with the highest risk observed in the 2012–2015 birth cohort (Figure 6). These changes may be related to changes in the national fertility policy: the single two-child policy in 2013, the two-child policy in 2016, and the three-child policy in 2021. Because the reported incidence of rotavirus infection in young children was very high, especially in children aged 0–2 years, and changes in fertility policies have changed the birth rate, the corresponding incidence risk across periods and cohorts has been affected.

Our study revealed that in 2022, the total rotavirus vaccination rate among hospitalised children aged 2–59 months in Zhejiang Province was 44.28% (Figure 7). This was lower than the rotavirus vaccination rate (46.80%) for children under 36 months in Guangzhou in 2013 (40), indicating that further improvement, such as including rotavirus vaccination in China’s national immunization plan, is needed to increase rotavirus vaccination coverage. The protective effect of RV5, which covers the G1, G2, G3, G4, and P (8) genotypes (41, 42), was consistent with the results reported in various other countries and our findings (43–45). Although the predominant circulating strain changes yearly, there is cross-protection (15) between genotypes. Hence, the efficacy of RV5 is naturally greater than monovalent vaccines. In this study, we found that after the intervention of RV5, the ASRIR of rotavirus infection decreased at a rate of 10.70/100,000 per year, and the reported average monthly number decreased by 3,061 cases (Figure 6). Thus, we suggest the inclusion of RV5 in immunization plans, especially in regions with high incidence rates of rotavirus infection, to increase rotavirus vaccination rates and reduce the disease burden of rotavirus infection.

This study had several limitations. First, rotavirus-related diarrhoea is a self-limiting disease, and some patients, especially adults, do not seek medical attention, are not tested, and are not diagnosed because of asymptomatic infection or mild symptoms; thus, the reported incidence of rotavirus diarrhoea obtained by surveillance may differ from the actual incidence rate. Second, the number of children surveyed regarding the rotavirus vaccination ratio of hospitalized children aged 2–59 months with diarrhoea was small, and only data from 2022 were obtained. These results do not represent the rotavirus vaccination rate in Zhejiang Province well. In the future, we will conduct more investigations to comprehensively and reliably assess the effectiveness of rotavirus vaccines against rotavirus infection. Third, modelling the expected incidence rate in this study did not account for changes in meteorological or sociological factors, such as average temperature, sunshine duration, wind speed, GDP per capita, or population demographics. We will refine the model after obtaining relevant data for more accurate conclusions. The ITSA model established in this study focuses on the intervention effect of RV5 on the reported incidence of rotavirus infection. Additionally, as mentioned in the study, rotavirus-related diarrhea is a self-limiting disease. Some mildly infected patients, may not seek medical attention, undergo testing or receive a diagnosis due to asymptomatic infections or mild symptoms. This leads to a potential disparity between the reported incidence of rotavirus diarrhea obtained from surveillance and the actual incidence. In future research, we will conduct community-based surveys, expand the surveillance network, use statistical models to estimate unreported cases, and explore novel diagnostic methods to better account for mild unreported cases. Moreover, we implicitly assumed that vaccinated individuals followed the recommended vaccination schedule without deviation. However, in actual real-world situations, vaccination compliance is a complex and variable factor. For future research, we will focus on collecting and integrating data related to vaccination compliance and will take it into account during the analysis. Besides, we acknowledge the limitations in our study regarding the lack of information on the number of vaccine doses administered and the potential mixing of different brands. In this study, our primary focus was on estimating the overall vaccine effectiveness and understanding the epidemiological trends of rotavirus infection. Due to the retrospective nature of the data collection and the existing data sources, we were unable to obtain detailed information on the number of vaccine doses and brand-mixing situations. For future research, we will design prospective studies with more comprehensive data collection protocols. Still, the novel coronavirus pneumonia epidemic at the end of 2019 may have affected the reported incidence. The implementation of non-pharmaceutical interventions (NPIs) during the COVID-19 pandemic likely had a significant impact on rotavirus transmission (46). Since rotavirus is transmitted through close contact and contaminated surfaces, the adoption of NPIs could have led to a decrease in the number of rotavirus infections (47). In future research, we will develop more sophisticated statistical models that can disentangle the effects of the pandemic from other confounding factors. Finally, in the analysis of the age-period-cohort model, missing data for 2023 and 2024 limited the analysis to data from 2020 to 2023, which may lead to the loss of some valuable information.

In future research, several promising areas can be explored. Emerging novel rotavirus vaccines in development, which aim to enhance immunogenicity and coverage by targeting additional rotavirus genotypes not well-covered by existing vaccines (48), should be investigated for their effectiveness, safety, and immunogenicity. Additionally, combination vaccines that include rotavirus antigens along with those against other common pediatric pathogens, like norovirus or adenovirus which cause diarrhea (49), could simplify vaccination schedules and potentially increase compliance. Finally, universal vaccination strategies need further exploration. Based on these insights, more effective immunization strategies can be developed to boost vaccination rates.

## Conclusion

Our research indicated that the disease burden of rotavirus infection in Zhejiang Province was high, especially in children. Rotavirus vaccines have significantly reduced the incidence rate of rotavirus infection. Therefore, the prevention of infectious diarrhoea should be further strengthened, especially coverage with the rotavirus vaccine.

## Data Availability

The original contributions presented in the study are included in the article/Supplementary material, further inquiries can be directed to the corresponding author.

## Footnotes

^1^ https://analysistools.cancer.gov/apc/

## Group member of RIDPHE Group

Innovation group on intelligent response to infectious diseases and public health emergencies. Shigui Yang, Xudong Zhou, Peige Song, Ning Zhang, Hao Lei, Junfang Xu, Jianbing Wang.
