# Supplementary material for: Epidemic characteristics and effectiveness of vaccine intervention on rotavirus infection: a real-world observational study in Zhejiang Province, China
Source: Front Public Health. 2025 May 9;13:1596899. doi: 10.3389/fpubh.2025.1596899 (PMC12098449; doi:10.3389/fpubh.2025.1596899)
Supplement: Supplementary file 1 [file Table_1.docx]

**Epidemic characteristics and effectiveness of vaccine intervention on rotavirus infection: A real-world observational study in Zhejiang province, China**

Ziping Miao^1†^, Yuxia Du^2†^, Anqi Dai^2†^, Mengya Yang^3†^, Can Chen^2^, Rui Yan^4^, Jian Gao^5^, Yijuan Chen^1^, Kexin Cao^2^, Daixi Jiang^2^, Xiaobao Zhang^2^, Xiaoyue Wu^2^, Mengsha Chen^2^, Yue You^2^, Wenkai Zhou^2^, Dingmo Chen^2^, Jiaxing Qi^2^, Shiyong Zhao^6^, Xianyao Lin^6^, Shigui Yang^2*^, RIDPHE Group^&^

**Supplementary materials**

Supplementary Material 1. Data sources and data information..................................3-6

Supplementary Material 2. Epidemiological trend analysis...........................................7

Supplementary Material 3. Spatial and temporal aggregation analysis ........................8

Supplementary Material 4. Interrupted time series analysis.....................................9-10

Supplementary Material 5. The correlation of rotavirus infection incidence rate in different age groups......................................................................................................11

Supplementary Material 6. The heat-map of reported incidence rate of rotavirus infection in different age groups from January 2005 to December 2022. The color bar indicates the intensity of incidence,counts from high (orange) to low (white). Monthly incidence counts were standardized for each year and shown as the proportion of the maximum number of cases in a month for that period (hence, months with the maximum number of cases for a given season were assigned the value 1)..................................................................................................................................12

Supplementary Material 7. Interrupted time series analysis on the reported incidence rate of rotavirus infection in different age groups in Zhejiang province from 2005 to 2022..............................................................................................................................13

Supplementary Material 8. Case enrollment flow chart for rotavirus vaccine effectiveness analysis ..................................................................................................14

Supplementary Material 9. General character..............................................................15

References....................................................................................................................16

**Supplementary Material 1 Data sources and data information**

**Data sources and data information**

In this study, we obtained data from three sources. The first data source was China Disease Prevention and Control Information System, which provided individual cases data of confirmed cases of rotavirus infection in all age groups among other reported cases of infectious diarrhea in Zhejiang Province from 2005 to 2022. By calculating the number of individual cases, the total number of cases in the corresponding year and age group was obtained. And the second data source was Zhejiang Provincial Bureau of Statistics [1], which provided the total population of Zhejiang province and each city from 2005 to 2022, so that to calculate the reported incidence rate of rotavirus infection. The reported incidence rate of rotavirus infection (per 100,000) was calculated as the number of reported cases of rotavirus infection divided by the population of the corresponding area, and the population data was obtained from the National Statistical yearbook [2]. The reported age-standardized reporting incidence rate (ASRIR) of rotavirus infection was calculated by the direct standardization method.

The formula was:

$p=\sum N*p_{i}$,

N was the standard age-specific population composition ratio; p_i_ was the age-specific incidence rate.

The p_i_ was calculated as described above. The standard age-specific population composition ratio data were also from the National Statistical yearbook.

Finally, the last data source was the Viral diarrhea surveillance site in Zhejiang province. This study comprehensively considered socio-economic factors, rotavirus infection monitoring and laboratory testing capabilities, as well as regional representativeness. Eight monitoring points were selected in counties (cities, districts) throughout the province, with one to two medical institutions selected as monitoring point hospitals for each monitoring point. From January 1, 2022 to December 31, 2022, rotavirus pathogen monitoring and related vaccination information investigation was conducted on diarrhea patients seeking medical treatment in the pediatric or digestive departments.

**Cases definition and inclusion criteria for monitoring objects:**

This study defined diarrhea as having bowel movements ≥3 times a day, accompanied by changes in stool characteristics (such as loose or watery stools), vomiting occurs ≥ 2 times within 24 hours. Infectious diarrhea is a group of infectious diseases caused by pathogenic microorganisms, their products or parasites, with diarrhea syndrome as the main clinical feature. And other infectious diarrhea only refers to infectious diarrhea except for cholera, dysentery, typhoid fever, and paratyphoid fever. The diagnosis of rotavirus-associated infectious diarrhea cases in this study was based on the "Diagnostic criteria and principles of management for infectious diarrhea" (GB17012-1997) and "Diagnostic criteria for infectious diarrhea" (WS271-2007): Cases with positive specific antigens or specific nucleic acid fragments were detected from feces and vomitus samples of diarrhea cases. Finally, the inclusion criteria for the monitoring subjects in this study were children≤ 59 months old who have diarrhea symptoms during hospitalization or hospitalization due to diarrhea, who meet the definition of diarrhea and have been hospitalized for≥ 24 hours, and children with secondary diarrhea symptoms caused by drugs or primary diseases were not excluded.

**Cases information collection and specimen testing:**

Medical staff from sentinel hospitals who have undergone unified training screened and monitored cases from departments such as internal medicine, pediatrics, and infectious diseases. The number of cases that meet the monitoring criteria would be determined according to the total outpatient/emergency cases of each sentinel hospital in the previous year. For the monitoring objects who meet the inclusion criteria,the unified "Diarrhoea Case Information Questionnaire" was used by special personnel to collect case demography (gender, age, occupation, address) and clinical information (onset date , visit date, symptoms, laboratory examination and treatment). Collected 5 grams of fecal samples from enrolled cases using sterile sampling cups (without adding preservation solution) and immediately stored them at -20 ℃, the storage time was less than 48 hours. The collected samples were transported to the network laboratory by a dedicated person for quality inspection and case information verification, as well as for group A, B, and C rotavirus testing and positive sample genotyping. The virus detection adopted real-time RT-PCR for nucleic acid amplification detection, using the reagent kit of Shanghai Zhijiang Biotechnology Co., Ltd. The operation method and result determination were carried out according to the requirements of the manual.

The Disease Prevention and Control Center in the jurisdiction where the sentinel hospital located was responsible for collecting vaccination information for hospitalized children with diarrhea under the age of 5 who were included in the monitoring. The collected information included the names, doses, and dates of each dose of rotavirus vaccines and other vaccines administered simultaneously. The vaccination information of hospitalized children with diarrhea under the age of 5 who could not be found due to cross regional visits would be supplemented by the superior disease prevention and control center. The above procedures were approved by the Ethics Review Committee of the Zhejiang Provincial Center for Disease Control and Prevention (Approval number: 2022-041-01).

**Supplementary Material 2. Epidemiological trends analysis**

**Epidemiological trends analysis**

The joinpoint regression model was used to examine the reported incidence trends of rotavirus infection by age (0-2 years old, 3-5 years old, 6-19 years old, 20-59 years old, and ≥ 60 years old) and gender from 2005 to 2022. The annual percentage changes (APCs) and their 95% confidence interval (CI) were obtained for each trend segment [14]. Z test was used to assess whether APCs were significant (P < 0.05), and the trends of increasing or decreasing reported incidence rate of rotavirus infection were further described based on whether statistically significant APCs are positive or negative. When the APCs values were not significant (P ≥ 0.05), the trends of the reported incidence rate were considered stable. The analysis was conducted by Joinpoint (version 4.9.0.0) [15] .

**Supplementary Material 3. Spatial and temporal aggregation analysis**

**Spatial and temporal aggregation analysis**

We used the spatiotemporal scan statistic, performed by SatScan (version 9.5), to explore the space-time cluster of reported rotavirus infection in Zhejiang province [21]. The dynamic space-time two dimensional cylinder scanning window was constructed to scan each location (city) within the study area, the size of the window was determined by the percentage of the city's total population size. The actual and theoretical incidence numbers inside and outside the scanning window were used to calculate the log likelihood ratio (LLR). The cluster was classified according to the LLR value [22] and the window with the maximum LLR value was determined as the first clustering area, followed by the secondary clustering area. The risk of disease in the cluster area was quantified as RR, which represents the probability of disease occurrence within the scanning window as a multiple of that outside the window. The Monte Carlo simulation was used to evaluate whether the cases are randomly distributed (P < 0.05).

**Supplementary Material 4. Interrupted time series analysis**

**Interrupted time series analysis**

The interrupted time series analysis (ITSA) model was constructed to analyze the changes in the reported annual rotavirus infection incidence in Zhejiang province before (2005-2018) and after (2018-2022) the intervention with the human-bovine reassortant pentavalent vaccine, RV5.

Segmented regression was used to fit the ITSA model:

$$Y_{t}=\beta_{0}+\beta_{1}time+\beta_{2}intervention+\beta_{3}post$$

Where, $Y_{t}$ was the time series data, representing the annually reported incidence of rotavirus infections in Zhejiang province from 2005 to 2022. $\beta_{0}$ was the intercept term; $\beta_{1}$ represented the trend of annually reported incidence of rotavirus infections before the RV5 intervention; $\beta_{2}$ meant the change in horizontal intercept caused by RV5 intervention, and also represented the recent impact of RV5 intervention on annually reported incidence of rotavirus infection $\beta_{3}$ was the amount of slope changes before and after RV5 intervention; ${(\beta}_{1}+\beta_{3})$ was the changing trend of the annually reported incidence rate of rotavirus infection after RV5 intervention. Time was a continuous time series, with values of 0, 1, 2, etc. The intervention was encoded as 0 before the RV5 intervention and 1 after the RV5 intervention. Post was assigned as 0 before the RV5 intervention, and then as 1, 2, etc after the RV5 intervention [23, 24].

In addition, we estimated the expected monthly incidence rate of reported rotavirus infection after the implementation of the RV5 intervention in September 2018 by using an over-dispersed Poisson model. Assuming that Y(t)~Poisson(µ(t)), where Y(t) represented the monthly reported rotavirus infection incidence, the model was:

$$\mu(t)=N(t)exp\{\beta t+g(w_{t})\}$$

The expected number of reported cases per month (t) was expressed by µ(t); N(t) was an offset that accounts for the population size; $\beta$ represented the linear effect of time, which explained the slow change of the incidence rate; $g(w_{t})$ was a function that explains seasonal trends, where $w_{t}$∈{1, 12} represented a month of the year [25, 26].

The ‘prais’ R package and ‘excessmort’ R package in RStudio were used to achieve the fitting and analysis of the above models.

**Supplementary Material 5. The correlation of rotavirus infection incidence rate in different age groups**


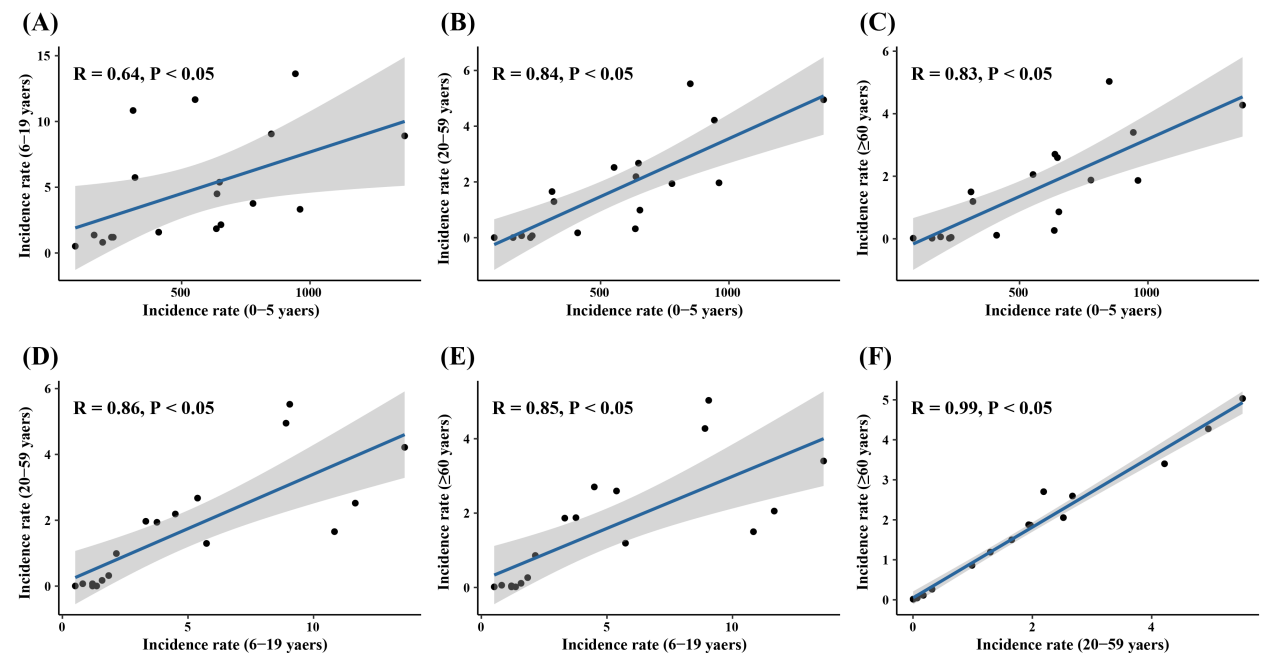


(A) 0-5 years old and 6-19 years old; (B) 0-5 years old and 20-59 years old; (C) 0-5 years old and ≥ 60 years old; (D) 6-19 years old and 20-59 years old; (D) 6-19 years old and ≥ 60 years old; (F) 20-59 years old and ≥ 60 years old.

**Supplementary Material 6. The heat-map of reported incidence rate of rotavirus infection in different age groups from January 2005 to December 2022.
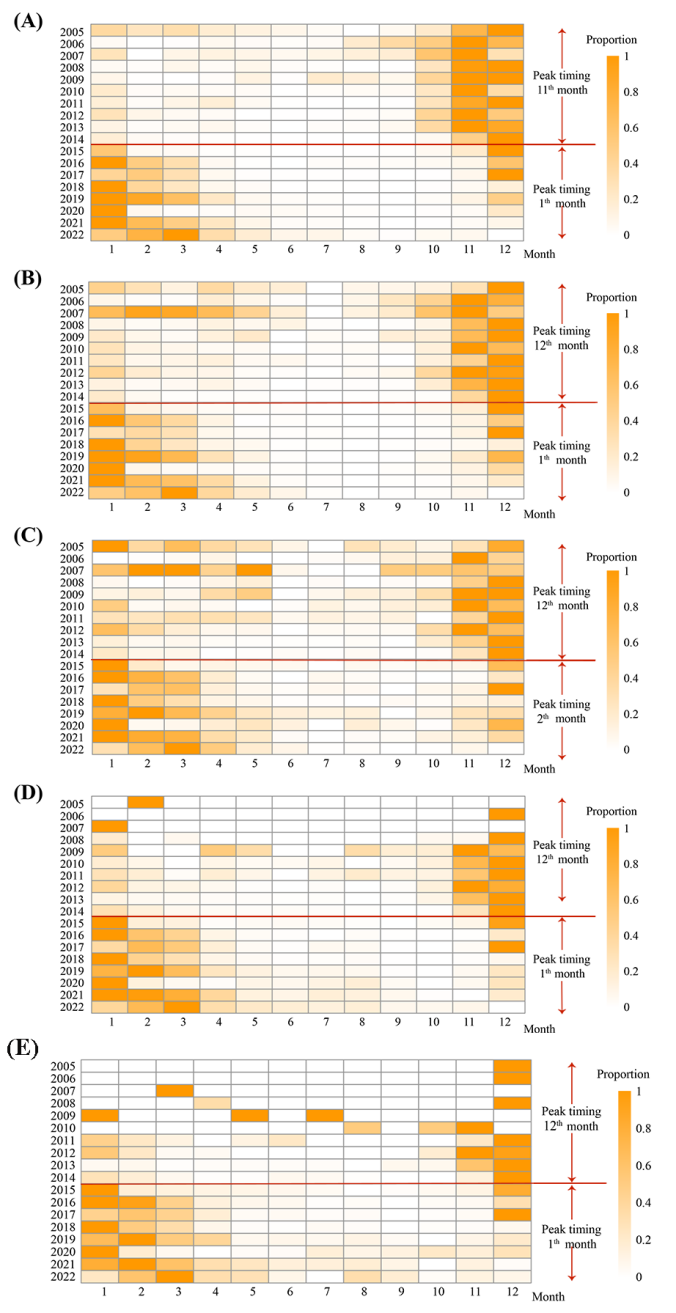
**

The color bar indicates the intensity of incidence, counts from high (orange) to low (white). Monthly incidence counts were standardized for each year and shown as the proportion of the maximum number of cases in a month for that period (hence, months with the maximum number of cases for a given season were assigned the value 1). (A) 0-2 years old. (B) 3-5 years old. (C) 6-19 years old. (D) 20-59 years old. (E) ≥60 years old

**Supplementary Material 7. Interrupted time series analysis on the reported incidence rate of rotavirus infection in different age groups in Zhejiang province from 2005 to 2022**


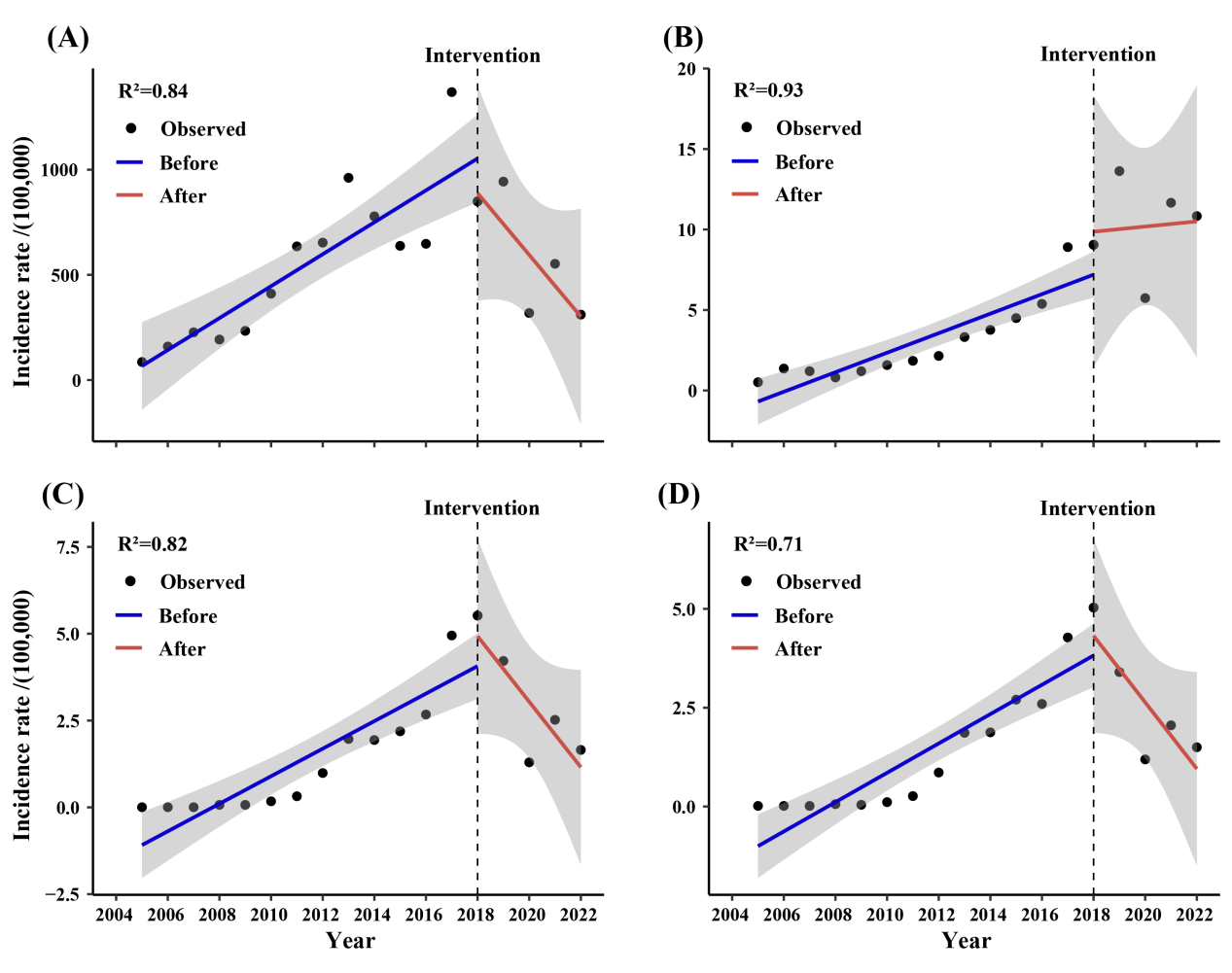


(A) 0-5 years old; (B) 6-19 years old; (C) 20-59 years old; (D) ≥ 60 years old.

**Supplementary Material 8. Case enrollment flow chart for rotavirus vaccine effectiveness analysis**

**
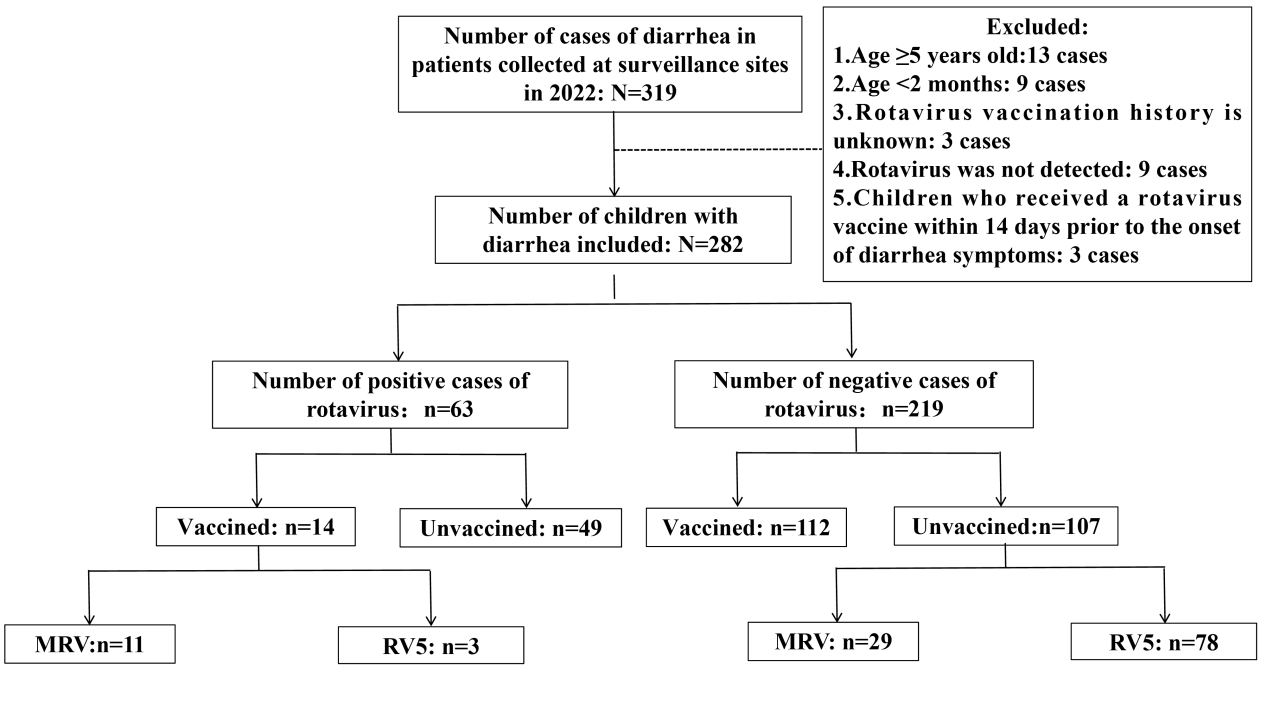
**

MRV: Monovalent rotavirus vaccine; RV5: human-bovine reassortant pentavalent vaccine.

**Supplementary Material 9.** **General character**

| **Groups** | **Case（n=63）** | **Control（n=219）** | ***P* value** |
| --- | --- | --- | --- |
| Age（month） | 19（12-33） | 17（10-30） | 0.24 |
| Sex |  |  | 0.74 |
| Male | 35（55.56%） | 129（58.90%） |  |
| Femal | 28（44.44%） | 90（41.10%） |  |
| Area |  |  | <0.05 |
| Binjiang District, Hangzhou | 47（74.60%） | 87（39.73%） |  |
| Gongshu District, Hangzhou | 12（19.05%） | 75（34.25%） |  |
| Huzhou | 0（0.00%） | 28（12.79%） |  |
| Lishui | 4（6.35%） | 29（13.24%） |  |
| Vaccination status |  |  | <0.05 |
| vaccinated | 14（22.22%） | 107（48.86%） |  |
| unvaccinated | 49（77.78%） | 112（51.14%） |  |
| Type of vaccination |  |  | <0.05 |
| Monovalent rotavirus vaccine | 11（78.57%） | 29（27.10%） |  |
| human-bovine reassortant pentavalent vaccine | 3（21.43%） | 78（72.90%） |  |

Values are presented as median (IQR) or number (%).

**References:**

1. Zhejiang Provincial Bureau of Statistics. http://tjj.zj.gov.cn/col/col1525563/index.html. Accessed on 28 March 2023.
2. China Statistical Yearbook-2022. http://www.stats.gov.cn/sj/ndsj/2022/indexch.htm. Accessed on 28 July 2023.
